# Prevalence of psychological symptoms in Saudi Secondary School girls in Abha, Saudi Arabia

**DOI:** 10.4103/0256-4947.55308

**Published:** 2009

**Authors:** Khalid S. Al Gelban

**Affiliations:** From the Department of Family and Community Medicine, King Khalid University, Abha, Saudi Arabia

## Abstract

**BACKGROUND AND OBJECTIVES:**

Adolescence is characterized by rapid physiological, social and cognititive changes. Aim of the present work is to study mental health of Saudi adolescent secondary school girls in Abha city, Aseer region, Saudi Arabia.

**METHODS:**

A cross-sectional study was conducted in 10 secondary schools for girls using the Arabic version of the symptom-revised checklist 90 (SCL 90-R), a mental health questionnaire that was administered to the girls by fourth-year female medical students.

**RESULTS:**

The most prevalent mental symptoms in the 545 female students were phobic anxiety (16.4%), psychoticism (14.8%), anxiety (14.3%), and somatization (14.2%). The prevalence of depression, paranoid ideation and interpersonal sensitivity amounted to 13.9%, 13.8% and 13.8%, respectively. The least prevalent mental symptoms were hostility (12.8%) and obsessive-compulsive behavior (12.3%). Overall, psychological symptoms (in terms of a positive global severity index) were found in 16.3% of the girls. In a multivariate logistic regression analysis, no significant relationship was found with sociodemographic factors.

**CONCLUSION:**

Psychological symptoms and disorders are prevalent in secondary school girls and health professionals need to be able to recognize, manage and follow-up mental health problems in young people. Further research is needed to explore the magnitude of the problem at the national level.

Adolescence (11 to 19 years of age) is characterized by rapid physiological, social and cognitive changes.^1^ Poor mental health during this period has been linked to mental health problems in adulthood.^2^ Worldwide, about 16% of adolescents suffer from mental health problems.^3^ In the United States, the direct cost of mental health services accounted for 69 billion US dollars, which represents 7.3% of total health spending.^4^ However, mental health promotion may help to prevent a wide range of health-damaging behavior as well as improving the quality of life of young people.^5^ In the United States, about 20% of children and adolescents are estimated to have mental health disorders.^6^ In Canada, 9.5% of boys and 12% of girls have mental health problems.^7^ Reports from India have estimated the prevalence of mental disorders from 2.6% to 35.6%.^8^ In the neighboring country of the United Arab Emirates, 22.2% of children aged 6 to 18 years were reported to have mental health problems.^9^ A large proportion of the population of Saudi Arabia is young, with 69% under 30 years old, and 47% under 15 years.^10^ However, there is a general paucity of research in the area of adolescent mental health. A recent study of boys in secondary school (age range from 15-18 years) in Abha city reported that more than one-third (38.2%) had depression, while 48.9% had anxiety and 35.5% had stress.^11^ Like other countries, adolescent girls in Saudi Arabia face rapidly changing challenges in their social and physical environment.^5^,^7^–^9^ Therefore, the purpose of this study was to estimate the prevalence of mental health symptoms among secondary school girls in Abha City in southwest Saudi Arabia and to assess related sociodemographic characteristics.

## METHODS

This cross-sectional study was conducted during the 2006-2007 scholastic year in Abha. The subjects were girls in secondary school (representing the tenth, eleventh and twelfth years of general education in Saudi Arabia, which starts at 7 years of age). Abha is the capital city of the Aseer region in the southwest of Saudi Arabia, with a total population of 300000. In Saudi Arabia, schools for girls are segregated from boys.

The city has a total of 10 secondary schools for girls, including 7 general schools and 3 Islamic (Tahfez Al-Quran) schools. All information about the study was provided to schools through the Abha School Health unit. All schools agreed to participate in the study. Field activities were conducted by trained fourth-year female medical students who were supervised by the staff of the Family and Community Medicine Department, College of Medicine, King Khalid University. Two classes were randomly selected from each school (first, second and third year).

The purpose of the study was explained to the participants in the classrooms. All students were clearly informed that they could choose not to participate. Subjects were assured of anonymity and confidentiality. All students in the selected classes during the study period were considered for inclusion in the study. Signed informed consent was given by each participant student.

The mental health questionnaire was based on the Arabic validated version of the Symptoms Check List 90-revised (SCL 90-R) questionnaire.^12^ This scale is used extensively to screen for mental health symptoms.^13^ The SCL 90-R test is a self-reporting instrument intended to measure severity of psychiatric symptoms on a number of different subscales. It contains 90 items and can be completed in just 12 to 15 minutes. Students rated the 90 symptoms of distress on a 5-step Likert-scale with 0 being ‘not at all’ and 4 being ‘extremely’. Subjects were instructed to indicate the amount they were bothered by each of the symptoms during the preceding week. Relevant dimension scores were summed and the ‘t’ transformation of the scores was performed. The positive dimension was considered when relevant *t* scores were 61 or more. The test helps in measuring nine primary symptom dimensions and three global indices of distress. It is designed to provide an overview of symptoms and their intensity at a specific point in time. The dimension includes somatization (the process by which psychological needs are expressed in physical symptoms), obsessive-compulsive (a form of personality marked by obsessions and compulsions), interpersonal sensitivity (relating to a conflict in the relations and social exchanges between persons), depression (a mental state of depressed mood characterized by feelings of sadness, despair and discouragement), anxiety (the unpleasant emotional state consisting of psychophysiological responses to anticipation of unreal danger), hostility (tendency to feel anger toward and to seek to inflict harm upon a person or group), phobic anxiety (fear that is recognized as being excessive or unreasonable by the individual himself), paranoid ideation (persistent delusions of persecution or delusional jealousy and behaviour such as suspiciousness, mistrust and combativeness), and psychoticism (a person will exhibit some qualities commonly found among psychotics such as disregard for common sense).^14^ The three global indices are Global Severity Index (GSI), Positive Symptom Distress Index (PSDI) and Positive Symptom Total (PST). GSI is designed to measure overall psychological distress. PSDI is designed to measure the intensity of symptoms. The PST reports the number of self-reported symptoms.

Statistical analysis was conducted using the SPSS version 15. All tests were carried out at the 5% level of significance. A multivariate logistic regression analysis was used to identify potential sociodemographic risk factors that might affect mental symptoms. Adjusted odds ratio and concomitant 95% confidence intervals were calculated.

## RESULTS

Five hundred and forty-five Saudi secondary school girls were included in the present study (Table 1). Their ages ranged from 14 to 19 years with a mean (SD) of 17.1 (1.1) years and a median of 17 years. Twenty students (3.7%) were married. Their ages ranged from 16 to 19 years. The majority (89.0%) had parents living together. The rest had separated (0.2%), divorced parents (4.2%) or a deceased father or mother (6.6%). The most frequent level of education among fathers was a university education (39.4%), while 7.9% were illiterate. On the other hand, the mothers' education was most often primary (29.9%), followed by none (28.3%). The most frequent occupation of fathers was as a military or governmental employee (46.0%). Most mothers were not working (87.9%). The majority of students (74.35%) were from families whose family size ranged from 5 to 10 persons. The average monthly family income ranged from 5000 to 10000 Saudi Riyals (SR) (1 US dollar equals 3.75 SR) in 47.5% of the study sample. The remainder were either below 5000 or above 10 000.

**Table 1:** Sociodemographic characteristics of the secondary school girls.

	No	%
**Age**		
14-15	22	4
16-17	333	61.1
18-19	190	34.9
**Marital status**		
Married	20	3.7
Single	525	96.3
**Scholastic year**		
First year	219	40.2
Second year	163	29.9
Third year	163	29.9
**Type of study**		
Oslamic (Tahfez Al-Quran)	209	38.3
General	336	61.7

**Parents' status**		
Living together	485	89.0
Divorced	23	4.2
Separated	1	0.2
Deceased father	28	5.1
Deceased mother	5	0.9
Both parents are deceased	3	0.6

**Father's education**		
Illiterate	43	7.9
Primary	87	16.0
Intermediate	93	17.1
Secondary	107	19.6
University	149	27.3
Postgraduate	66	12.1

**Father's employment**		
Unemployed	25	4.60
Military	89	16.3
Governmental employee	162	29.7
Private business	106	19.5
Retired	163	29.9

**Mother's education**		
Illiterate	154	28.3
Primary	163	29.9
Intermediate	88	16.1
Secondary	69	12.7
University	54	9.9
Postgraduate	17	3.1
**Mother's employment**		
Housewife	479	87.9
Employed	66	12.1

**Family size**		
Less than 5	41	7.5
5-10	405	74.3
More than 10	99	18.2

**Total**	**545**	**100**

Table 2 shows the overall prevalence of mental symptoms among adolescent secondary school girls. The most frequent mental symptoms were phobic anxiety (16.4%), psychoticism (14.8%) and anxiety (14.3%). The least frequent dimensions were obsessive-compulsive behavior (12.3%) and hostility (12.8%). Some students had more than one symptom. Forty-nine girls (9.0%) had simultaneously 2 symptoms, 31 girls (5.7%) had 3 symptoms and 24 girls (4.4%) had 4 symptoms. Table 3 shows the global indices of distress. The positive global severity index (GSI) amounted to 16.3%, which indicates that at least one out of each of six girls suffers from one or more mental health symptoms. The positive symptoms distress index was 13.7% and positive symptoms total was 14.0%.

**Table 2:** Overall prevalencea of mental symptoms among secondary school girls in Abha, Saudi Arabia.

Mental symptom	Prevalence^a^	
	No	%
Phobic anxiety	90	16.5
Psychoticism	80	14.7
Anxiety	78	14.3
Somatization	77	14.1
Depression	76	13.9
Interpersonal sensitivity	75	13.8
Paranoid ideation	75	13.8
Hostility	70	12.8
Obsessive-compulsive behavior	67	12.3

a Categorization was based on “t” transformation of SCL R-90 scores. Positive dimension was considered when “t” scores are 61 or more.

^a^Total is more than 100% as some students have more than one disorder (comorbidity)

**Table 3:** Positive global indices of distress among secondary school girls in Abha, Southwestern of Saudi Arabia (n= 545).^a^

Positive global indices of distress	Prevalence (%)
Global Severity Index (GSI)	16.3
Positive Symptoms Distress Index (PSD)	13.7
Positive Symptoms Total (PST)	14.0

a Categorization was based on “t” transformation of SCR R-90 scores. Positive dimension was considered when “t” scores are 61 or more.

Table 4 shows multivariate logistic regression analysis of potential sociodemographic risk factors affecting the highest three prevalent mental symptoms: phobic anxiety, psychotocism and anxiety. None of the potential sociodemographic factors (age, type of school, marital status, family size, parental status, parental education and occupation and average of family income) were found to significantly affect the highest three prevalent dimensions of mental health. The same findings were present in the rest of the mental health dimensions.

**Table 4:** Adjusted odds ratio (aOR) and 95% confidence intervals (CI) for potential sociodemographic risk factors that might be associated with the highest three prevalent mental symptoms among secondary school girls in Abha, Saudi Arabia (n=545), by multivariate analysis.

Potential Socio-demographic Risk Factor	Phobic Anxiety	Psychotocism	Anxiety
Age (<17 years vs. ≥17 years)	1.1 (0.6-2.2)	1.1 (0.6-2.2)	1.5 (0.8-3.0)
School (general vs. culture)	0.8 (0.4-1.4)	0.8 (0.4-1.5)	0.6 (0.3-1.2)
Marital status (single vs. married)	0.9 (0.3-3.0)	0.8 (0.2-3.1)	1.8 (0.4-8.3)
Family size (<5 persons vs. ≥5 persons)	0.7 (0.3-1.6)	1.9 (0.6-5.9)	0.6 (0.3-1.4)
Parental status (living together vs. others)	0.7 (0.4-1.9)	1.4 (0.6-2.9)	1.8 (0.9-3.7)
Father education (illiterate vs. educated)	1.4 (0.6-3.3)	1.0 (0.4-2.6)	1.4 (0.6-3.6)
Mother education (illiterate vs. educated)	1.4 (0.8-2.4)	0.6 (0.3-1.1)	0.9 (0.5-1.7)
Father occupation (retired or not working vs. working)	0.8 (0.4-1.3)	1.1 (0.7-2.0)	0.7 (0.4-1.3)
Mother occupation (not working vs. working)	1.1 (0.6-3.3)	1.1 (0.5-1.2)	1.5 (0.8-3.0)
Average family income (<5000 SR vs. ≥5000 SR)	1.1 (0.6-1.9)	1.2 (0.7-2.2)	0.8 (0.4-1.5)

## DISCUSSION

Using a standardized instrument that assesses a broad range of mental health problems, we found that symptoms were prevalent among secondary school girls, with 16.3% suffering from one or more mental health symptoms.

In a previous study conducted on secondary school boys in the same area using Depresion, Anxiety and Stress Scale (DASS) questionnaire, indicated that of 1723 Saudi adolescent school boys, 59.4% had at least one of the three studied disorders (depression, anxiety or stress), 40.7% had at least two and 22.6% had all three disorders. Moreover, more than one-third of the participants (38.2%) had depression, while 48.9% had anxiety and 35.5% had stress. Depression, anxiety and stress were strongly, positively and significantly correlated.^11^ Fehan et al reported that 63% of New Zealand adolescents have mental illness.^15^ Other studies found rates of 22.2% in United Arab Emirates,^8^ 18.5% in USA,^16^ 17.9% in Puerto Rico,^17^ 22.5% in Switzerland,^18^ 9% among boys and 12% among girls in Canada,^6^ 8% in India,^7^ and 20.3% in Taiwan.^19^ The prevalence ranged from 1% to 51% (mean=15.8%) conducted over the last 40 years from 20 countries.^3^ This variation could be related to using different measure tools.

The most frequent mental symptoms were phobic anxiety (16.4%), psychoticism (14.8%) and anxiety (14.3%), which is in agreement with the literature that indicates that anxiety disorders are the most common psychiatric diagnosis in adolescents.^20^,^21^ In an earlier study of 1723 male high school students in Saudi Arabia using an Arabic version of DASS, nearly 49% were found to have an anxiety disorder.^11^ Studies indicate that females have a higher prevalence of anxiety.^7^,^22^ On the other hand, the least frequent dimensions in this study were obsessive-compulsive (12.2%) and hostility (12.9%). In the UAE, Eapen et al reported that 7% and 9% of adolescents have phobia and anxiety, respectively.^8^ Fehan et al found that in New Zealand, anxiety and simple phobia were present in 65% and 41%, respectively.^15^

The majority of students came from families that ranged in size from 5 to 10 persons, which is in agreement with the average Saudi family size reported in previous research.^10^ About two-thirds of mothers had either a primary education (29.9%) or were illiterate (28.3%). Unlike our finding, Brandon found that children whose mothers are employed were more likely to have emotional problems which can be secondary to the differences. Average family income was less than 5000 SR in 21.8%. However, this did not significantly affect the nine dimensions of mental health. Neither did other potential sociodemographic risk factors (age, school type, marital status, family size, rank among brothers and sisters, parental status, parental education and occupation and average monthly family income (Table 4). A risk factor does not necessarily imply a direct causal relationship. It has been reported that emotional problems are most likely to be due an interaction between biological and environment factors.^24^ Integration of mental health into medical setting might improve care and reduce stigmatization of mental illness.^25^ Primary health care centers care offer a setting for the prevention and detection of mental health problems in adolescents. Further research is needed to determine the magnitude of psychiatric disorders at national level.

The questionnaire used in this study had the advantage of being psychometrically validated and developed in consideration of cross cultural and linguistic issues. However, this study has some limitations: it was restricted to female adolescents, which led to an inability to determine sex-specific psychopathology. Since it was a school-based study, it might miss adolescents in the community who do not attend schools. However, the results would reflect the state of psychopathology among adolescents.

In conclusion, the high prevalence of mental symptoms represents one of the major health problems affecting adolescents in Abha city. Further in-depth studies are needed to study mental disorders in the area and to identify potential supporting counseling and cognition-behavior therapy. In this context, a national program for mental health in this age group is crucial.
